# Fitness Loss and Library Size Determination in Saturation Mutagenesis

**DOI:** 10.1371/journal.pone.0068069

**Published:** 2013-07-03

**Authors:** Yuval Nov

**Affiliations:** Department of Statistics, University of Haifa, Haifa, Israel; UMR-S665, INSERM, Université Paris Diderot, INTS, France

## Abstract

Saturation mutagenesis is a widely used directed evolution technique, in which a large number of protein variants, each having random amino acids in certain predetermined positions, are screened in order to discover high-fitness variants among them. Several metrics for determining the library size (the number of variants screened) have been suggested in the literature, but none of them incorporates the actual fitness of the variants discovered in the experiment. We present the results of an extensive simulation study, which is based on probabilistic models for protein fitness landscape, and which investigates how the result of a saturation mutagenesis experiment – the fitness of the best variant discovered – varies as a function of the library size. In particular, we study the loss of fitness in the experiment: the difference between the fitness of the best variant discovered, and the fitness of the best variant in variant space. Our results are that the existing criteria for determining the library size are conservative, so smaller libraries are often satisfactory. Reducing the library size can save labor, time, and expenses in the laboratory.

## Introduction

Directed evolution has emerged as an indispensable vehicle for engineering biocatalysts, therapeutics, and other protein-based tools. Through iterative rounds of mutagenesis and selection that mimic natural evolution, directed evolution “breeds” proteins with improved or novel chemical properties [1]. A key concept in evolutionary biology is that of *fitness* – the capacity of an organism to survive and reproduce. This meaning of the term originates from the phrase “survival of the fittest,” coined by Herbert Spencer and adopted by Charles Darwin. John Maynard Smith extended the notion of the so-called “fitness landscape” of entire organisms to the molecular world of proteins [2], and more recent writers have used the term “fitness” in the context of protein engineering, to denote any desirable protein property that is subject to optimization [3]–[5]; we follow this usage below.

Though the very essence of directed evolution protocols depends on screening large numbers of mutants, recent works increasingly advocate relatively small, yet carefully designed libraries. Such targeted libraries enjoy a significantly higher fraction of functional mutants, compared to completely random libraries, and thus achieve remarkable experimental results. Various principles are used in the design of targeted libraries, including reduced amino-acid alphabets [6], inference on ancestral sequences [7], and combinatorial spiking [8]; for a recent review, see Goldsmith and Tawfik [9]. In this work we provide another argument in favor of smaller libraries – one which is related to an experimental procedure called saturation mutagenesis.

Saturation mutagenesis is a simple *in vitro* directed evolution technique, whereby the amino acids at certain preselected positions along a protein sequence are replaced by random ones, in a manner called *randomization*. A large number of the resulting mutants are generated to form a library, and are then screened, in the hope of discovering among them a highly improved variant of the wildtype protein. Saturation mutagenesis has been used successfully to improve a host of desirable protein properties, including thermostability, specificity, catalytic activity, enantioselectivity, binding affinity, and photostability [10]–[14]. The method can also be used iteratively, to probe efficiently larger portions of protein space [15].

All randomization schemes for saturation mutagenesis originate at the DNA level. Most commonly, the three wild-type nucleotides at each randomized codon are replaced with random ones. If all four nucleotides are allowed at each of the codon’s three positions – a randomization scheme known as NNN – then all 4^3^ = 64 possible codons may be formed. Other randomization schemes use reduced codon sets, and include NNB, NNS, and NNK (where N = A/C/G/T, B = C/G/T, S = C/G, K = G/T), which are all superior to NNN randomization as they induce a lower probability of introducing a premature stop codon, as well as a more uniform probability distribution across the twenty amino acids [16]. Still better schemes eliminate completely, or almost completely, both codon bias and the stop codon problem; this can be achieved either by replacing entire wild-type codons by random ones [17], or as shown recently, by using an appropriately weighted mixture of primers [18], [19].

Regardless of the chosen randomization scheme, the larger the library, the higher the likelihood of exploring more distinct protein variants at the screening stage, and hence the higher the probability of discovering a novel, high-fitness variant. On the other hand, generating and screening large libraries is laborious, time consuming, and expensive. To balance judiciously the benefits and drawbacks associated with the library size, one needs a *metric* – a performance measure that can be calculated before the actual fitness of the library’s variants is observed – and understand how this metric depends on the library size.

Two well known such metrics are: (i) the probability that all possible variants are represented in the library (“probability of full coverage”); and (ii) the expected percentage of all possible variants that are represented in the library (“expected coverage”). Exact and approximate mathematical expressions for these two metrics can be found in [20]–[22].

Recently, another metric has been proposed: the probability that the library contains at least one of the *k* top-performing variants (in terms of their fitness), for some small integer *k* [23]. The probability that the library contains the best variant – i.e., the case *k* = 1 – is clearly a meaningful metric; under very mild assumptions on the distribution of fitness, this case can be shown to be the same as metric (i) above. The rationale behind this metric for *k* ≥2 is that often, especially when the number of all possible distinct variants is very large, there are several variants whose fitness satisfies the design requirements, and discovering any of these top-performing variants will be considered a success. Using a metric of the latter type, even with *k* as low as 2, allows the designer to reduce significantly the library size, and thus to save laboratory resources and labor.

However, none of the above metrics incorporates directly the actual fitness of the variants to be discovered in the process, which is arguably the most relevant metric. The goal of this work is to fill this gap, and to study *in silico* how the fitness of the best variant discovered in a saturation mutagenesis experiment varies as a function of the library size. We wish to emphasize that it is not the intention of this work that protein engineering experimentalists should repeat themselves any of the computational or mathematical procedures described below (running simulations, estimating parameters, etc.); rather, we aim to derive general guidelines that are applicable to a wide range of saturation mutagenesis experiments, and that can be used by those practitioners.

To attain this study’s goal, one needs a model for “protein landscape” – the relationship between a protein’s sequence and fitness. This relationship is notoriously complex, and indeed, several attempts have been made to model it mathematically. We use for this purpose the probabilistic approach, i.e., view the sequence–fitness relationship as a realization of a *random* phenomenon, so that the fitness of each variant, before its exact value is revealed in an experiment, is only known to have a certain distribution. Among the probabilistic models for the sequence–fitness relationship are Kauffman and Weinberger’s *NK* model [24], Aita and Husimi’s “rough Mount Fuji” model [25], the model of Nov and Wein [26], and machine-learning techniques such as Fox et al.’s ProSAR [27].

Our main modeling vehicle in this work is the aforementioned model of Nov and Wein; this model was devised specifically for protein design purposes, and was used successfully to improve the reduction activity of the *E. coli* enzyme ChrR [28], and the oxidizing activity of the toluene-4-monooxygenase (T4MO) enzyme [29]. To ground our simulation results in experimental data, we use below the parameters estimated in these studies, as well as those estimated from another empirical work [30]. For further support of our conclusions and as a control, we also use the *NK* model and the “rough Mount Fuji” model.

## Methods

### Probability

Let the randomized protein positions be labeled *i* = 1,…., *M*, and let *Q_a_*_,*i*_ be the probability that the randomization will result in amino acid *a* at position *i*. The probabilities *Q_a_*_,*i*_ are induced by the randomization scheme and the genetic code: for example, under NNN randomization at position *i*, since 4 out of the 64 codons encode alanine, *Q*_Ala,*i*_ = 4/64. The probability that the randomization will result in a nonsense mutation (a premature stop codon) at the codon corresponding to amino acid *i* is *Q*_stp,*i*_ = 1 − ∑*_a_ Q_a_*_,*i*_; depending on the randomization scheme, this probability may be zero or positive.

Denote by *A_i_* the number of amino acids with positive probability to appear at position *i*, i.e., *A_i_* = |{*a* : *Q_a_*_,*i*_ > 0}|. Note that *A_i_* < 20 in some randomization schemes [6], [31]. We use the term *variant space* to denote the set of all possible distinct protein variants that may be formed in the experiment; the number of elements in this space is *n* = *A*_1_ *A*_2_…*A_M_*. Assuming that the randomizations across the *M* positions are independent of each other, the probability that a random variant will have amino acid *a*_1_ in randomized position 1, amino acid *a*_2_ in randomized position 2, etc., is 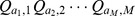. Let *p*_1_,…, *p_n_* be an enumeration of these product probabilities, corresponding to the *n* distinct variants in variant space. The probability that a random variant will have at least one nonsense mutation, and will thus be completely dysfunctional, is 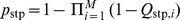.

Let *L* be the library size, i.e., the number of protein variants whose fitness is measured, let *X_v_*, *v* = 1,…, *n*, be the number of copies of variant *v* that are included in the library, and let *X*_stp_ be the number of variants with a nonsense mutation. Then the random vector (*X*_1_,…, *X_n_*, *X*_stp_) has a multinomial distribution with parameters *L*, *p*_1_,…, *p_n_*, *p*_stp_. Denote by *V* the (random) subset of variant space that is represented in the library: *V* = {*v* : *X_v_* ≥ 1}. In this notation, the probability of full coverage is *P*(|*V*| = *n*) and the expected coverage is *E*(|*V*|/*n*).

Let *F_v_* be the fitness of variant *v*. We denote by *v* ^*^ the variant with the highest fitness discovered in the experiment (i.e., the best among those that happened to be represented in the library), and by *v* ^**^ the globally fittest variant in variant space (i.e., the best among all distinct variants that could possibly be generated in the experiment). Formally,
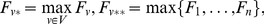
(1)


There are two sources of randomness contributing to the variability of 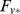. The first is the aforementioned randomness of the set *V*, which is *experimental* in origin, i.e., stems from the randomness of the set of variants chosen by the experimenter to constitute the library. The second is the randomness of the mapping *v* 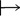 *F_v_* (the protein landscape), which is *natural* in origin: even when the sequence of a variant is known, its fitness is determined by the laws of chemistry in such an intricate way, that we resort to model it probabilistically. Only the second type of randomness plays a role in the randomness of 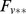. While the first type of randomness requires only elementary probability to model and analyze, the second, as stated earlier, is more intricate and challenging to study. Three approaches to modeling the relationship between sequence and fitness are outlined in the next subsection.

Assuming that fitness is a continuous characteristic, so that there are no fitness ties, and assuming that no variant may be considered *a priori* to be better than another, the distribution of the fitness ranking (from best to worst) across variant space is uniform over all *n*! permutations of its elements. Let *T_k_* be the event “the library contains at least one of the top *k* variants in variant space,” and let 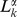 be the minimal library size required to ensure a probability α of discovering at least one of the top *k* variants, i.e., 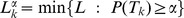. See [23] for methods to compute *P*(*T_k_*) and 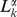.

### Sequence–fitness Models

#### The model of nov and wein

The model of Nov and Wein [26] aims to capture the following three characteristics of the sequence–fitness relationship:

- **Wild-type dominance**. Mutations in the wild type are more likely to decrease fitness, rather than to increase it, and the more mutations a variant has, the lower is its resulting fitness, on average [32]–[35].
- **Clustering**. Given a favorable mutation at a certain position, other mutations at this position are more likely to be favorable as well. Indeed, the basic rationale behind the saturation mutagenesis approach is that certain “hotspots” are more likely than others to accommodate favorable mutations [36].
- **Additivity**. The change in fitness due to multiple mutations equals approximately to the sum of the corresponding individual fitness changes [37]–[39]. Importantly, additivity may be weak when measured using the raw fitness values, but can become stronger if the measurements are transformed to a different scale; see below.

In this model, the raw fitness values are transformed so that the wild type, denoted by 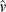, has zero fitness: 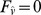. The fitness of any other variant 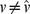 is viewed as a realization of a random variable *F_v_*, and the collection of the random variables (*F*_1_,…, *F_n_*) is a realization of a random vector whose distribution is governed, in a manner described below, by four parameters: the drift *m*, the position variance 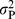, the amino-acid variance 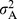, and the non-additivity variance 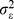.

A mutation at position *i*, which replaces the wild-type amino acid 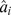 with another amino acid *a*, contributes a random quantity 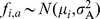 to the fitness of *v*. The *M* means μ_1_,…, µ*_M_* are not constants, but rather, independent random variables themselves (in a way resembling the random effect model from statistics), having a 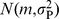 distribution, where *m* < 0. The fitness of a variant *v* that has amino acid *a*_1_ in position 1, amino acid *a*_2_ in position 2, etc., is then given by
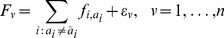
(2)where ε_1_,…, ε*_n_* are 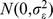 random variables, independent of each other, of the *f_i_*_,*a*_’s, and of the µ*_i_*’s.

By setting the drift *m* to be negative, the model captures the “wild-type dominance” characteristic. By having the 19 random variables 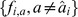 at each position *i* share a common random mean µ*_i_*, we establish a positive correlation among them, which captures the clustering characteristic. Finally, the additive structure of (2) captures the additivity characteristic, with 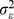 determining its magnitude.

It can be shown that the resulting collection of random fitness variables (*F*_1_,…, *F_n_*) is a multivariate Gaussian process, and that for two variants *v* and *v*′, having respectively the amino acids 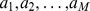 and 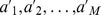 at the randomized positions, this process satisfies
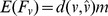


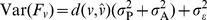
(3)

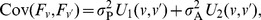
where 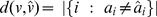 is the Hamming distance between *v* and 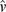 (the number of positions in which *v* and the wild type differ); 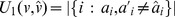 (the number of positions in which both *v* and *v*′ differ from the wild type); and 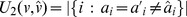 (the number of positions in which *v* and *v*′ have the same mutation).

For transforming the raw fitness values into the values *F_v_*, we use in this work mainly the logarithmic transformation (relative to the wild type), in which a raw fitness *x* is transformed by 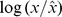, where 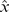 is the wild-type’s raw fitness. The scaling by 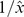 guarantees that 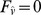, as required by the model. This logarithmic transformation was used in the two empirical studies involving the model of Nov and Wein [28], [29].

The four parameters of the model used below were estimated using the maximum likelihood approach: Given a vector of fitness observations 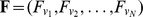 for *N* variants 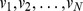, the estimated parameters are those that maximize the likelihood of **F**, i.e., that solve the maximization problem
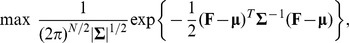
where 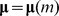 and 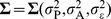 are the mean vector and covariance matrix of 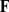, respectively, whose entries satisfy equation (3). See [26] for more details about this estimation techniques, and [28], [29] for the method used to address sampling bias problems.

### The *NK* Model

The *NK* model, introduced by Kauffman and Weinberger [24], is a spin-glass-like model of random epistatic interactions, conceived to study evolutionary adaptive walks in protein space. According to this model (and using its original notation, which is different from that used in the previous subsection), the total fitness of a protein variant is the average 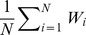 of the fitness contributions *W_i_* from the protein’s *N* positions. The fitness contribution of each position is a *U*(0, 1) random variable that depends not only on the amino acid at that position, but also on the amino acids at *K* < *N* other positions.

The value of *K* determines the ruggedness of the protein landscape: When *K* = 0, the fitness contributions from the *N* positions are independent of each other, there is a strong positive correlation between the fitness of neighboring variants (i.e., variants whose sequences differ by a single amino acid), and the resulting smooth landscape contains a single peak. At the other extreme, when *K* = *N* − 1, the fitness values of all 20*N* variants in protein space are independent of each other, resulting in a highly rugged landscape with many local peaks.

The *K* positions influencing the fitness contribution of each position *i* may be either randomly chosen, or the nearest *K* positions flanking position *i* in the primary sequence. We follow the simulations of Kauffman and Weinberger, and use the latter option.

### The Rough Mount Fuji Model

According to the “rough Mount Fuji” model of Aita and Husimi [26], and using its original notation, the fitness of a variant having sequence P is the sum *W*_P_+ω_P_, where *W*_P_ is an additive term and ω_P_ is a non-additive term. The non-additive term is modeled as a *N*(0, ϱ^2^) random variable, and the additive term is the sum of the fitness contributions from the protein’s *ν* positions: 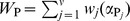, where 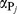 is the *j*th amino acid in the sequence P, and the fitness contribution of amino acid α*_i_* at position *j* is *w_j_*(α*_i_*) = ε*i*/10, *i* = 0, 1,…, 19. The parameter ε < 0 is the mean fitness contribution resulting from placing an “incorrect” amino acid (i.e., *i* ≠ 0) at each position.

By placing the “correct” amino acid (*i* = 0) at each of the *ν* positions, the additive term *W*_P_ attains its maximal value, zero. However, the final fitness of a variant having only “correct” amino acids (i.e., with 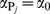 for all *j*) may not be the global optimum, because of the non-additive terms ω_P_. The ratio |ε|/ϱ is a measure of smoothness of the fitness landscape, with low values corresponding to a rugged landscape and high values corresponding to a smooth one.

### Computation and Simulation

The 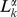 values and the “full coverage” probabilities were computed by a C computer program called TopLib; see stat.haifa.ac.il/∼yuval/toplib for documentation. The simulations involving the sequence–fitness models were coded in the R programming language (The R Foundation for Statistical Computing, www.r-project.org); each simulation was based on 5000 replications. When using the model of Nov and Wein, the simulated landscapes were initially generated following equation (2) and then exponentially transformed, so that the resulting values need to be logarithmically transformed to fit the model.

## Results

The goal of this work is to study the relationship between the library size *L* and the loss of fitness in a saturation mutagenesis experiment, i.e., the difference between the fitness of the globally fittest variant, 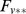, and the fitness of the best variant discovered in the experiment, 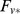 (see (1)).

Both 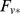 and 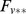 depend on the sequence–fitness model used, and in particular, on the model’s parameters; 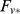 (but not 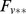) depends also on the randomization scheme (NNN, NNK, etc.). We now explore, via computer simulations, how the loss of fitness depends on *L* under the various models and parameters.

### Studying Loss of Fitness

**Figure 1:**
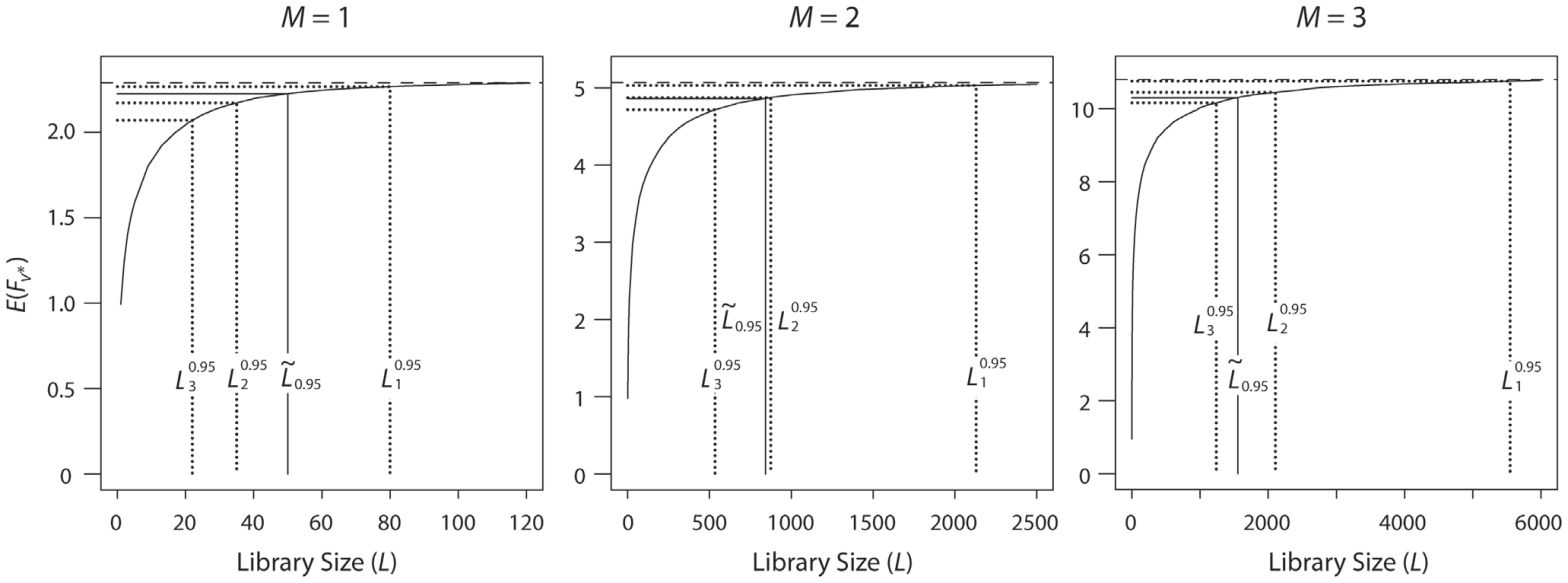
Expected fitness as a function of library size. Expected fitness of the best variant discovered in the experiment, 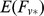, as a function of the library size *L*, when randomizing *M* = 1, 2, 3 NNK positions. The horizontal dashed lines denote the expected fitness of the best variant in variant space, 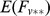. The dotted lines correspond to the library sizes required to ensure a 0.95 probability of discovering the top variant (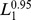), any of the top two (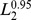), and any of the top three (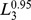). The solid line correspond to 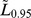, the library size required to reach 95% of the distance from the lowest point of the curve (at *L* = 1) to 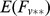. Simulations are based on the model of Nov and Wein, with the T4MO parameter and a logarithmic transformation.

We first study how 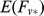 converges to 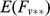 as *L* increases. Figure 1 depicts this convergence under NNK randomization of *M* = 1, 2 and 3 positions; the underlying probabilistic model is that of Nov and Wein, with the parameters estimated in the T4MO study [29]: the variance parameters are 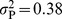, 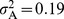, 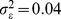, and the drift parameter was taken to be the median of the values used in the study, *m* = −0.3. Still in accordance with that study, it is assumed that the raw activity values need to be logarithmically transformed in order to meet the model’s probabilistic assumptions.

In each of the three panels of Figure 1, the horizontal upper dashed line intersects the vertical axis at 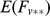, and thus serves as an upper bound for the curve of 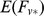. The three dotted lines correspond to 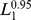, 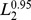, and 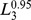, i.e., to the library sizes required to ensure a 0.95 probability of discovering the best variant (rightmost line), any of the top two variants (middle), and any of the top three (left). The solid line corresponds to a library size which we denote by 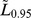: the size required to reach 95% of the distance from the expected fitness of a single randomized variant (i.e., the lowest point of the curve, corresponding to *L* = 1) to 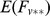. This 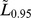 serves as a rough indication for a reasonable library size, which nearly exhausts, on average, the fitness that may be extracted from the fitness landscape.

It is evident that as *L* increases, the difference between 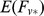 and 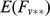 becomes negligible. For example, when randomizing *M* = 2 positions (middle panel of Figure 1), a library size of about 1,000 appears to be satisfactory: When 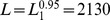 variants, we have 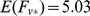, a loss of about 0.8% relative to 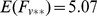, so the requirement *P*(*T*_1_) = 0.95 seems overly stringent. When 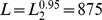 or 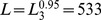, we have 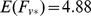 (3.7% loss) or 4.72 (6.9% loss), respectively. When 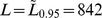, we have 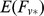 = 4.86 (4.1% loss), which indeed appears reasonable, though it is up to the experiment designer to decide what magnitude of loss is considered negligible. The library size required to achieve a 0.95 probability of “full coverage” (metric (i) mentioned in the Introduction) is 8,128 – almost an order of magnitude more than the ∼1,000 that appear to suffice, and is therefore extremely wasteful.

Figure 1 also shows that as expected, 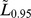 increases sharply in absolute numbers as the number of randomized positions increases, from 50 to 842 to 15,562, corresponding to *M* = 1, 2, 3, respectively. However, 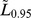 *decreases* relative to the landmarks 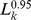: when *M* = 1, 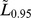 is about half way between 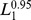 and 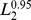; when *M* = 2, 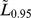 is close to 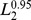; and when *M* = 3, 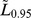 is about half way between 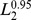 and 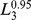.

For the remainder of this work we fix the number of randomized positions at *M* = 2, and study how the choice of model and its parameters influence the difference between 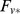 and 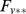, as a function of *L*.

**Figure 2:**
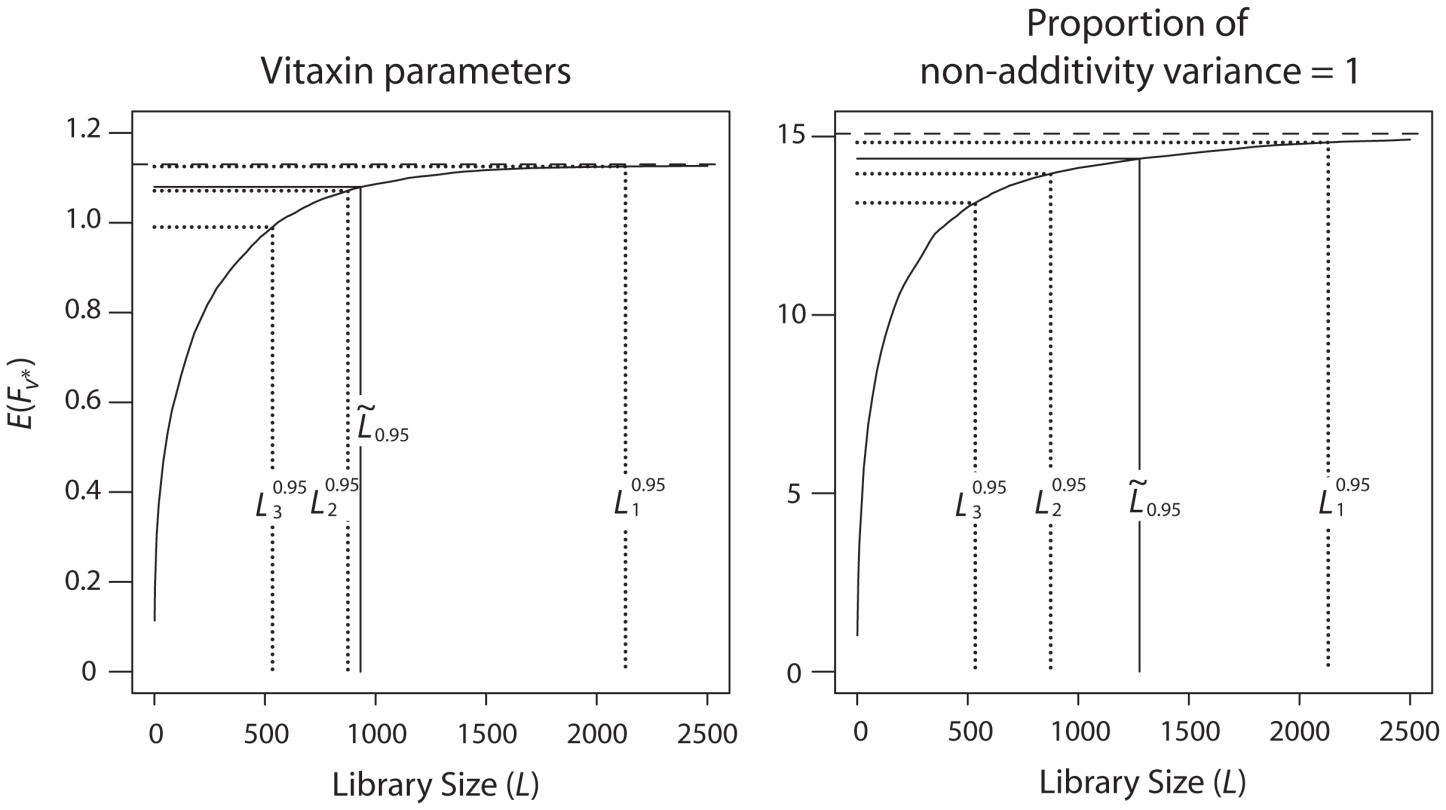
Expected fitness as a function of library size, other landscape parameters. Expected fitness of the best variant discovered in the experiment, 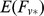, as a function of the library size *L*, when randomizing two NNK positions. Left: Vitaxin parameters. Right: same marginal fitness distribution as with the T4MO parameters, but the proportion of non-additivity variance equals 1.

The only other study in which all four parameters of the model of Nov and Wein were estimated is [26]. That study analyzed data which was used to improve the binding affinity of an α*_v_*β_3_-specific humanized monoclonal antibody called Vitaxin [30]. The estimated parameters are *m* = −1.35, 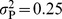, 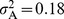, 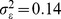, and the raw data were logarithmically transformed. The left panel of Figure 2 shows again the convergence of 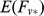 to 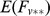 when randomizing two NNK positions, this time under the Vitaxin parameters. In this case, 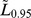 =  932, slightly above 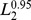 =  875. The main factor contributing tothis moderate increase in 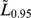, relative to the corresponding value under the T4MO parameters (middlepanel of Figure 1), is the higher proportion of the non-additivity variance from the total fitness variance:when randomizing two positions, this proportion is 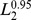 =  875. The main factor contributing tothis moderate increase in 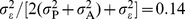 (see equation (3)) inthe Vitaxin parameters, whereas it is only 0.034 in the T4MO parameters. To demonstrate the influenceof high non-additivity variance, we repeated the T4MO simulation under the (unrealistic and extreme) parameterization in which this proportion is 1, without changing the marginal distribution of the fitnessvalues. This was done by keeping *m*  =  −0.3, setting 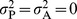, and letting 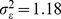, which is thefitness variance of an arbitrary variant (with two randomized positions) under the T4MO parameters.The results are shown in the right panel of Figure 2; as expected, 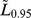 has significantly increased, to 1,277.It can be shown mathematically, using Slepian's inequality [40], that a high proportion of non-additivityvariance yields more conservative estimates for the library size, so that the right panel of Figure 2 is themost challenging scenario.

**Figure 3:**
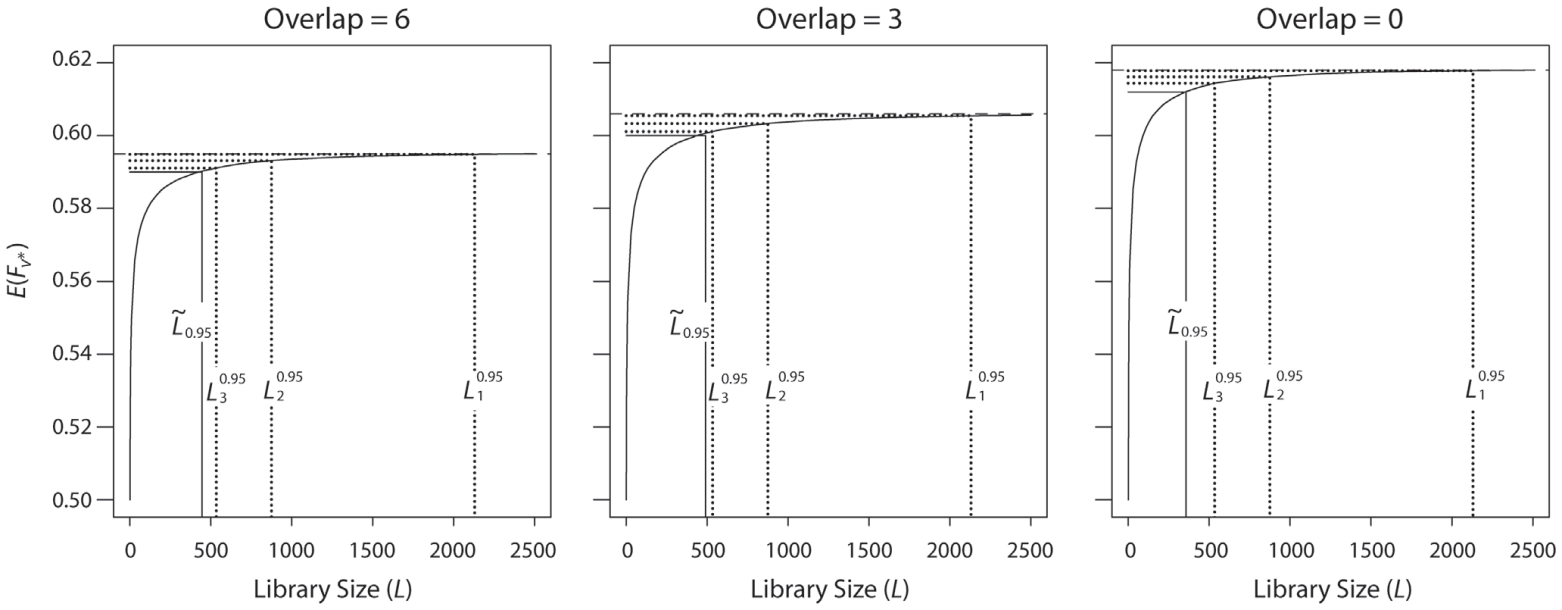
Expected fitness as a function of library size, the *NK* model. Expected fitness of the best variant discovered in the experiment, 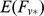, as a function of the library size *L*, when randomizing two NNK positions, under the *NK* model. Parameters are *N* = 24 and *K* = 6. The number of overlapping positions among those influencing the fitness contributions from the two randomized positions is 6, 3, and 0. Given *K*, it still remains to determine the degree of overlap between the *K* +1 positions influencing the first randomized position, and the *K* +1 influencing the second. We consider therefore three scenarios: maximal overlap of *K* = 6 positions, moderate overlap of 3 positions, and no overlap. The resulting values of 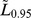 are 446, 491, and 358, respectively.

We now turn to modeling the fitness landscape by models other than the model of Nov and Wein. Figure 3 shows the results when randomizing two NNK positions using the *NK* model, with parameters *N* = 24 (the median value of the *N*’s used in the simulations in [24]) and *K* = 6 (the median value of the *K*’s for *N* = 24, in that study). Given *K*, it still remains to determine the degree of overlap betweenthe *K*+ 1 positions influencing the first randomized position, and the *K* + 1 influencing the second. Weconsider therefore three scenarios: maximal overlap of *K*  =  6 positions, moderate overlap of 3 positions,and no overlap. The resulting values of 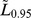 are 446, 491, and 358, respectively.

**Figure 4:**
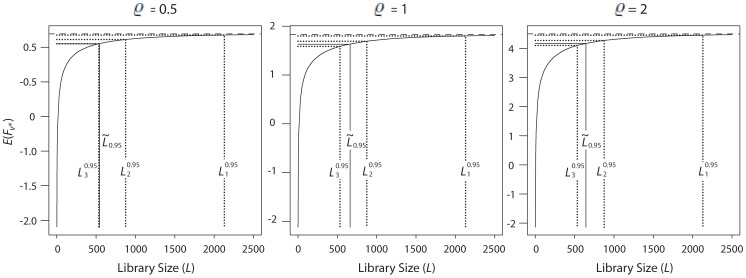
Expected fitness as a function of library size, the rough Mount Fuji model. Expected fitness of the best variant discovered in the experiment, 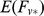, as a function of the library size *L*, when randomizing two NNK positions, under the rough Mount Fuji model. Parameters are ν = 60, ε = −1, and ϱ = 0.5, 1, 2.

Figure 4 shows the results when modeling the fitness landscape through the rough Mount Fuji model. The parameters are ν = 60, ε = −1, and ϱ = 0.5, 1, and 2 (the values used in [25]). The resulting valuesof 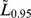 are 543, 663, and 644, corresponding to the three values of ϱ.

Thus, under both the *NK* model and the rough Mount Fuji model, 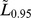 is significantly smaller than the value 842, obtained under the model of Nov and Wein, so the latter model is more conservative. All the results presented so far concerned only mean values. We define the percent loss of fitness to be.
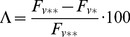
(4)which is a random variable taking values in the set [0, 100], and whose distribution strongly depends on the library size *L*. Clearly, lower values of Λ are desirable, with Λ = 0 corresponding to the (ideal) event 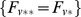.

**Figure 5:**
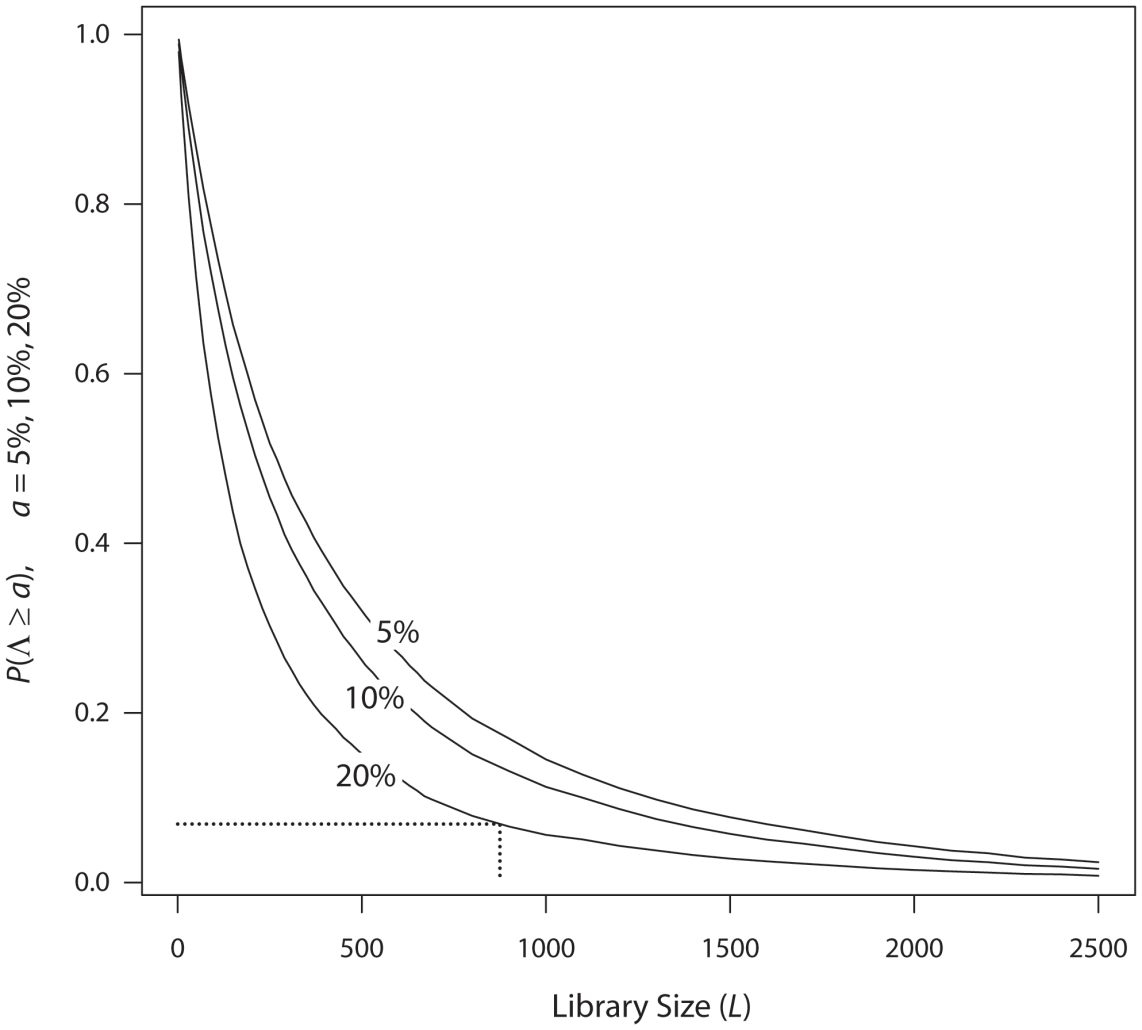
Probability of losing more than 5%, 10%, 20% of fitness. The probability that the fitness of the best variant discovered 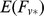 will be at least 5%, 10%, and 20% lower than that of the best variant in variant space 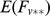. Randomization, fitness model, parameters, and transformation as in Figure 1.

Figure 5 shows the loss probabilities 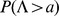 for 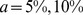, and 20% (under the model of Nov and Wein, T4MO parameters, and a logarithmic transformation). For example, when the library size is 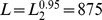, there is a 0.069 probability that the fitness of the best variant discovered will be lower than that of the best variant in variant space by more than 20% (the dotted lines).

**Figure 6:**
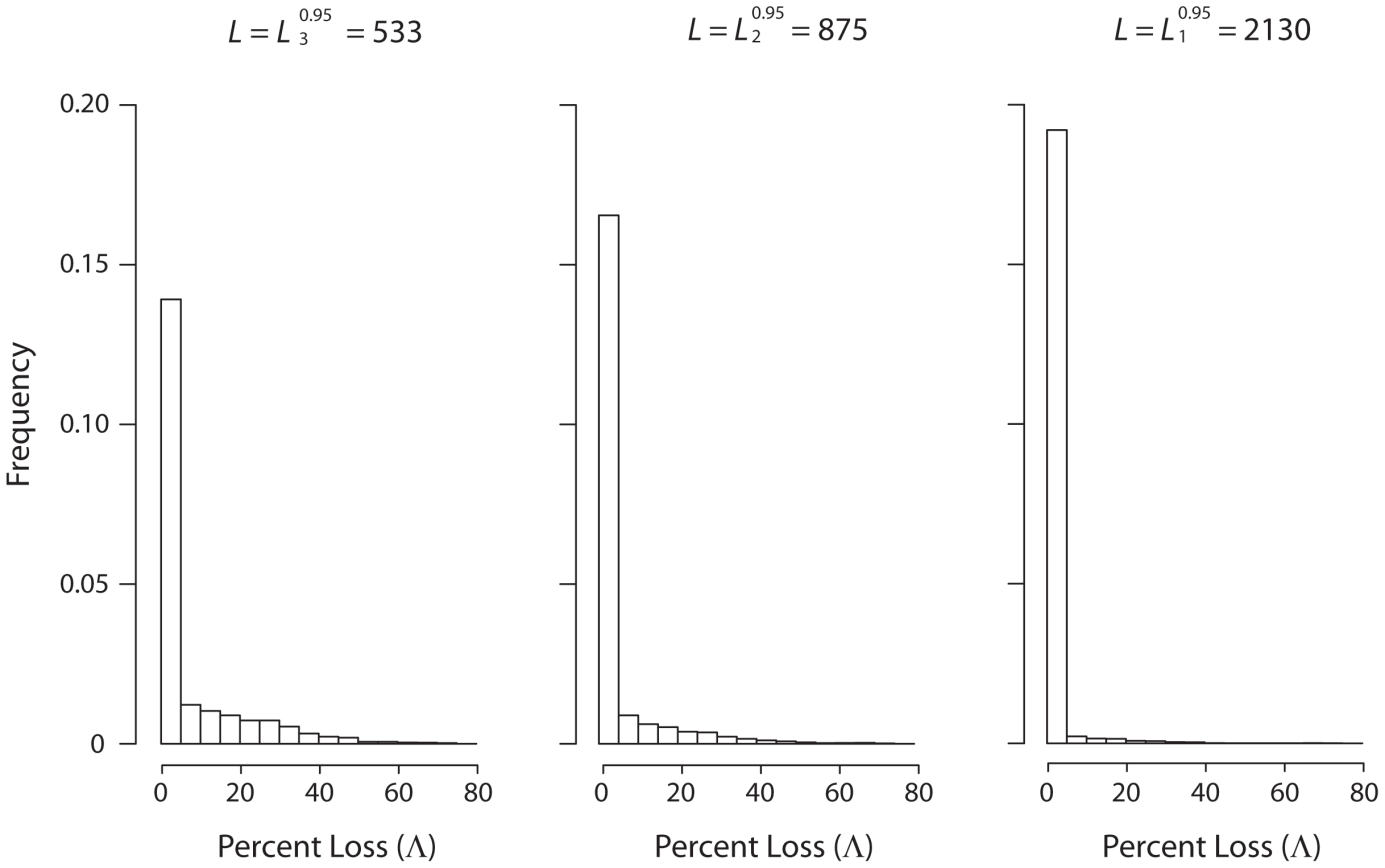
Distribution of loss of fitness as a function of library size. Distribution of the loss of fitness for three library sizes: 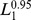, 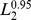, and 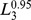. Randomization, fitness model, parameters, and transformation as in Figure 1.

Figure 6 shows histograms depicting the distribution of Λ for three library sizes: 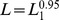, 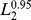, and 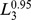. The spiking, leftmost column in each of the three histograms corresponds almost in its entirety to the case Λ = 0. As expected, the height of this column increases as *L* increases, and the distribution reflected by the remaining columns becomes stochastically closer to zero.

## Discussion

It is difficult to establish universal, sharp guidelines for the optimal library size in a saturation mutagenesis experiment, as the requirements from the design process, the screening costs, and the fitness landscape of the specific protein at hand vary from one experiment to another. However, judging from the results of the extensive simulation study presented above, it seems that when randomizing two positions, a rough yet reasonable rule of thumb is to choose a library size no larger than a point about half way between 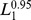 and 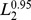, i.e., between the library size required to ensure a 0.95 probability of discovering the best variant, and that of discovering any of the top two variants. Using a similar methodology, one can establish similar rough guidelines for library size determination when randomizing *M* ≠ 2 positions. We have done so (results not shown) for the cases *M* = 1 and *M* = 3: When randomizing a single position, we recommend a library no larger than about 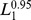 variants, and when randomizing three positions, about 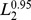 variants.

These recommendations are conservative, as they are based on the model that provides the largest library sizes among the three models considered (the model of Nov and Wein), and on the most challenging scenario from a wide range of model parameters (right panel of Figure 2).

In [29] it was suggested that the logarithmic transformation is perhaps “too concave” for converting the raw fitness measurements, and that to achieve a better fit to the model of Nov and Wein, a power transformation such as the Box–Cox transformation [41] with power 0 < λ ≤1 should be used instead (such transformations are used routinely in statistics to improve model fit, and λ has no physicochemical interpretation). However, since a Box–Cox transformation with 0 < λ ≤1 is “less concave” than the logarithmic transformation, the resulting library sizes will be smaller; thus, assuming that a logarithmic transformation is required, as was done in this work, is again conservative.

The library sizes we recommend are *lower* than those actually chosen by some practitioners, sometimes by a very large margin. In two unrelated experiments, each involving randomization of a single NNS position, Boersma et al. [42] screened 1,250 variants and Maeda et al. [43] screened 360 variants, where ∼80 variants seem to suffice. Similarly, Champion et al. [44] screened ∼8,000 variants when randomizing two NNN positions, where ∼2,000 seem to suffice.

One may use the approach described in this work to compare the efficiency of the various randomization schemes. We have done so, and the results are consistent with previously published findings [16], [23]: the most efficient schemes are those that induce a 1/20 probability for each of the amino acids [17], [18], next is NNK (or NNS, which is identical in this sense), then NNB, and the least efficient scheme is NNN.

Another approach to protein engineering, which often complements directed evolution, is rational design. Here, scientists use elaborate computational algorithms and detailed structural knowledge of the protein at hand, in order to introduce targeted mutations that are predicted to improve the protein's fitness. When taking a pure rational design approach, library size is minimal, as only the targeted mutants need to be generated via site-directed mutagenesis. Often, rational design and directed evolution are combined into the so-called semi-rational approach, whereby computational tools dictate which positions are best to be explored by saturation mutagenesis [45]. The library size analysis presented in this work is relevant to such experiments. Some rational design studies aim at designing highly stable, “ideal” protein structures; such structures may be used as scaffolds for the next generation of engineered proteins, over which saturation mutagenesis could be employed to focus the desired function [46].

### Conclusions

Our simulation results indicate that existing criteria and practices for determining the library size in saturation mutagenesis experiments are often too conservative: smaller libraries, sometimes by an order of magnitude, suffice to exhaust almost all the fitness that the fitness landscape has to offer. This observation is in line with other recent works, which advocate the use of “small but smart” libraries [9]. The reduction in library size carries clear benefits in laboratory expenses, time, and labor.
